# *Mycobacterium tuberculosis* Peptidyl-Prolyl Isomerases Are Immunogenic, Alter Cytokine Profile and Aid in Intracellular Survival

**DOI:** 10.3389/fcimb.2017.00038

**Published:** 2017-02-15

**Authors:** Saurabh Pandey, Deeksha Tripathi, Mohd Khubaib, Ashutosh Kumar, Javaid A. Sheikh, Gaddam Sumanlatha, Nasreen Z. Ehtesham, Seyed E. Hasnain

**Affiliations:** ^1^Inflammation Biology and Cell Signaling Laboratory, National Institute of PathologyNew Delhi, India; ^2^Department of Biology, Dr. Reddy's Institute of Life Sciences, University of HyderabadHyderabad, India; ^3^Molecular Infection and Functional Biology Laboratory, Kusuma School of Biological Sciences, Indian Institute of TechnologyNew Delhi, India; ^4^Bhagwan Mahavir Medical Research CentreHyderabad, India; ^5^Jamia Hamdard, Institute of Molecular MedicineNew Delhi, India

**Keywords:** bacterial survival, cyclophilins, *M. tuberculosis*, immune response, THP-1 cells

## Abstract

*Mycobacterium tuberculosis* (*M. tb*) has two peptidyl-prolyl isomerases (Ppiases) PpiA and PpiB, popularly known as cyclophilin A and cyclophilin B. The role of cyclophilins in processes such as signaling, cell surface recognition, chaperoning, and heat shock response has been well-documented. We present evidence that *M. tb* Ppiases modulate the host immune response. ELISA results revealed significant presence of antibodies to *M. tb* Ppiases in patient sera as compared to sera from healthy individuals. Treatment of THP-1 cells with increasing concentrations of rPpiA, induced secretion of pro-inflammatory cytokines TNF-α and IL-6. Alternatively, treatment with rPpiB inhibited secretion of TNF-α and induced secretion of IL-10. Furthermore, heterologous expression of *M. tb* PpiA and PpiB in *Mycobacterium smegmatis* increased bacterial survival in THP-1 cells as compared to those transformed with the vector control. Our results demonstrate that *M. tb* Ppiases are immunogenic proteins that can possibly modulate host immune response and enhance persistence of the pathogen within the host by subverting host cell generated stresses.

## Introduction

Tuberculosis caused by the intracellular pathogen *Mycobacterium tuberculosis* (*M. tb*), remains a potential threat regardless of strong efforts to alleviate its toll on humanity. Intracellular/intraphagosomal survival of this pathogen plays a critical role in the infection cycle of the pathogen, a process which majorly relies on array of virulence factors to colonize and replicate within the host macrophages (Ehrt and Schnappinger, 2009). Virulence factors which have been known to play a vital role in host pathogen interaction at the molecular level include stress responders, heat shock proteins (HSPs), foldases, and chaperones (Henderson, 2010). Studies involving immunological characterization of these effector molecules can bridge huge gaps in our understanding of *M. tb* biology and facilitate better therapeutic and diagnostic interventions.

Protein folding in the cell is assisted by molecular chaperones and foldases. The foldases generally include peptidyl-prolyl isomerases and protein disulfide isomerases. In addition to peptidyl prolyl isomerase activity, Ppiases have been shown to play role in diverse biological processes such as receptor signaling, apoptosis, stress response, and RNA-mediated gene expression (Sykes et al., 1993; Lu et al., 1996; Wu et al., 2000; Mark et al., 2001). Human cyclophilin A and also *M. tb* Ppiases have been reported to possess chaperone activity (Zhang et al., 2013; Pandey et al., 2016). Many such chaperones and HSPs have also been known to have immune modulatory role during bacterial infections. For example, mycobacterial HSP65 induces a strong cellular and humoral immune response (Peetermans et al., 1994; Friedland et al., 2008).

*M. tb* is known to possess two Ppiases (cyclophilins), PpiA and PpiB. *M. tb* PpiA is a part of the secretome and is known to interact with host proteins involved in immune defense mechanism and signal transduction (Henriksson et al., 2004; Bhaduri et al., 2014) while PpiB has been reported in membrane fraction and mannosylation enriched culture filtrate (Cole et al., 1998; Gu et al., 2003). Immunological characterization of these enzymes in terms of their possible role in modulating host immune response, virulence and intracellular survival, has not been investigated so far. PpiB, being an essential protein (Sassetti et al., 2003), makes it an important target for developing new interventions.

In the present study, we describe the immunogenic potential of *M. tb* cyclophilins, their involvement in eliciting host immune response, altering the host cytokine profile and promoting the intracellular survival of the pathogen, significant attributes which highlight the seminal role of these proteins in the infection process of *M. tb*.

## Materials and methods

### Materials

IPTG, imidazole, α-chemotrypsin, reduced Glutathione, N-succinyl-Ala-Ala-Pro-Phe-p-nitroanilide, trifluoroethanol, LiCl, DTT, H_2_O_2_, and polymyxin B were purchased from Sigma. Cell culture reagents were purchased from GIBCO, Thermo Fisher Scientific (USA). All restriction enzymes were purchased from New England Biolabs (USA); antibodies from Abcam (UK); and ELISA kits from Peprotech (USA). Mycobacterium growth media and supplements were purchased from Becton, Dickinson and Company (USA). All reagents used were analytical grade. The strains and plasmids used in this study are listed in Table S1.

### Mycobacterial strains

*Mycobacterium smegmatis* mc^2^155, initially obtained from ATCC, was maintained in our laboratory as glycerol stocks. Running culture was obtained by inoculating in 7H9 broth supplemented with 10% OADC and 0.05% Tween 80. Cultures were incubated at 37°C in a shaker incubator for suspension cultures. For CFU enumeration, bacteria were plated on 7H10 Middlebrook agar plates supplemented with 10% OADC and incubated at 37°C. Genomic DNA of *M. tb* H_37_Rv used in the cloning was kind gift from Dr. Manjula Sritharan and Dr. Sharmishtha Banerjee, University of Hyderabad, Hyderabad, India.

### Enzyme assay of purified recombinant Ppiases

The genes encoding *M. tb PpiA* (Rv0009) and *M. tb PpiB* (Rv2582) were PCR amplified from genomic DNA of *M. tb* strain H_37_Rv, using forward and reverse primers and cloned in pET28a and pGEX6p-1 vector as described (Pandey et al., 2016). Recombinant proteins were purified (Banerjee et al., 2007) using Ni-NTA column for PpiA and glutathione sepharose affinity column for PpiB. Ppiase activity of both, rPpiA and rPpiB was evaluated using a spectrophotometric assay (Pandey et al., 2016).

### Antigenicity profiling

Antigenic index of PpiA and PpiB was analyzed *in silico* using protein analysis software (Protean version 4.0, Lasergene Navigator; DNA STAR Inc; Madison, Wis; Chakhaiyar et al., 2004).

### Human subjects

This Study was approved by Institutional Bioethics Committee, and written consent was obtained from all participants. The two categories recruited for the study were: fresh confirmed cases of pulmonary TB (*n* = 43) and healthy control (*n* = 43). Subjects reporting to hospital with symptoms of tuberculosis were selected on the basis of sputum smear positivity. Healthy controls were the volunteers with no symptom or history of TB. HIV^+^ individuals were excluded from the study. Human blood samples were collected and processed as described earlier (Tundup et al., 2008). Briefly, the blood was withdrawn by venipuncture of median cubital vein by a phlebotomist. Isolated blood was allowed to clot for half an hour at 37°C. It was then centrifuged at 1500 × g for 15 min to remove the clot. The clear serum was aliquoted and stored at −80°C until needed.

### Immune assays

The antibody levels in human serum were assayed by enzyme linked immunosorbent assay (ELISA) using microtiter plates (Corning) coated with rPpiA, and rPpiB respectively, as described earlier (Banerjee et al., 2004; Khubaib et al., 2016). Briefly, 96 well plates were coated by specific proteins in PBS (10 μg/ml) and kept at 4°C overnight. Plate was washed three times with wash buffer and blocked for an hour at room temperature. After three washes, serum samples in 1:100 dilutions were added and kept for 2 h. Secondary conjugate antibody (1:10,000) was added for an hour. Plate was washed at least five times, TMB substrate was added, and reaction was stopped with 2N H_2_SO_4_.

Ppiase induced secretion of cytokines from macrophages was also quantified by ELISA as described earlier (Silswal et al., 2005; Nair et al., 2009). Differentiation of THP-1 cells was achieved by phorbol 12-myristate 13-acetate (PMA) treatment (Nair et al., 2009). Differentiated cells were plated and treated with appropriate concentration of respective proteins. Supernatants were harvested at various time points and quantified for determination of various cytokine levels. Briefly, 96 well ELISA plates were coated with capture antibody in coating buffer (bicarbonate/phosphate buffer) kept at 4°C overnight. After an hour of blocking with assay diluent, supernatants along with standards were added for 2 h. Enzyme conjugates were then added for 1 h. TMB substrate was added and 2N H_2_SO_4_ was added to stop the reaction. Absorbance was measured at 450 nm and curve was plotted along with standards to determine the cytokine levels in test samples. All the usual steps of intermittent washings were included as per manufacturer's instructions.

### Cloning, expression of *M. tb* Ppiases in *M. smegmatis*

Cloning and expression of *M. tb* Ppiases in *M. smegmatis* was carried out as described (Farhana et al., 2008; Tripathi et al., 2015) using *E. coli*-mycobacterium shuttle vector pST2K and specific oligonucleotide primers (Tables S1, S2). The resulting constructs, pST_ppiA, pST_ppiB and pST2K vector were then electroporated in wild type *M. smegmatis* (Ms_WT). Transformed recombinant *M. smegmati*s strains were designated as Ms_ppiA, Ms_ppiB, and Ms_VC (Table S1).

### *In vitro* growth and stress assay

Log phase cultures of *M. smegmatis* strains (Ms_WT, Ms_VC, Ms_ppiA, Ms_ppiB) were inoculated in Middlebrook 7H9 broth with 10% Oleic Albumin Dextrose Catalase (OADC) in the presence/absence of kanamycin (25 μg/ml). The cultures were grown at 37°C at 200 rpm. The cell density was measured periodically at 600 nm (OD_600_) using spectrophotometer. For hydrogen peroxide stress, log phase cultures (OD_600_ of 0.8–1.0) of *M*. *smegmatis* strains were diluted 1:100 into Middlebrook 7H9 broth and grown for approximately 12 h until the OD_600_ reached 0.4. Re-inoculated cells were then treated with the 7 mM H_2_O_2_. At 0, 1, 2, and 3 h, 100 μl samples were serially diluted and plated on Middlebrook 7H10 plates to determine the colony forming units (Li et al., 2014).

### Uptake and intracellular growth of *M. smegmatis* expressing *M. tb* Ppiases in human THP-1 macrophages

THP-1 monocytic cell line was cultured and differentiated with PMA in RPMI 1640 medium as reported earlier (Nair et al., 2009; Tripathi et al., 2015). The THP-1 monolayers were infected with log-phase bacteria (Ms_WT, Ms_VC, Ms_ppiA, and Ms_ppiB), for 4 h at MOI of 50. The cells after infection were treated with medium containing 20 μg/ml gentamicin for 30 min, and washed twice with RPMI medium. The plates were incubated at 37°C after adding fresh complete medium. The cells were dislodged gently at different time points and centrifuged at 2000 rpm for 3 min, washed twice with fresh RPMI 1640 medium and lysed in sterile water. The lysate was then diluted in Middlebrook 7H9 broth and plated on Middlebrook 7H10-OADC agar plates. The plates were incubated at 37°C and colonies were counted after 4 days. For each data point, the mean of triplicate was used.

## Results

### *Mycobacterium tuberculosis* Ppiases display antigenic stretches and also elicit significantly higher B-cell response in TB patients

**Figure 1:**
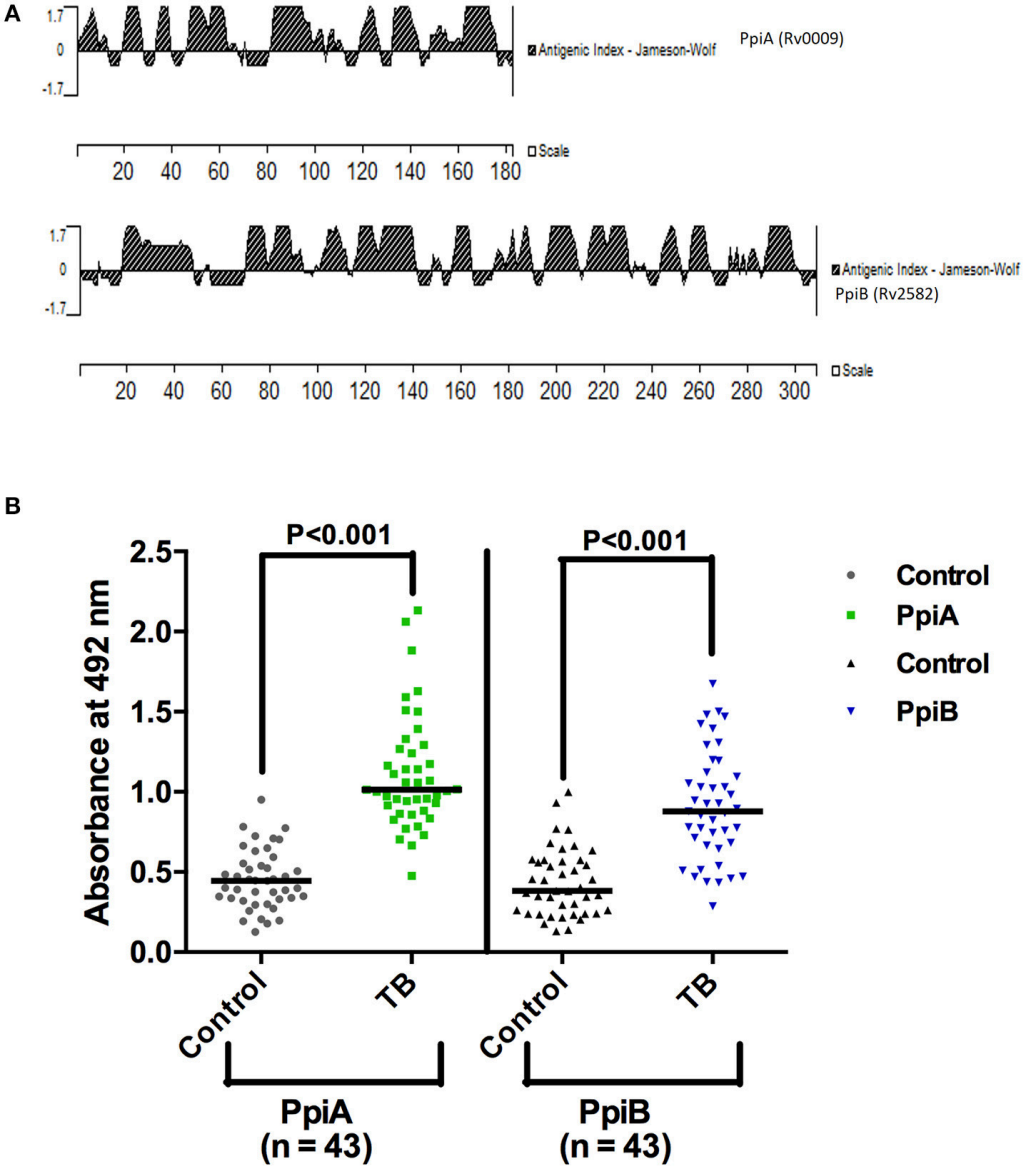
**Mycobacterial Ppiases elicit B cell response in tuberculosis patients. (A)** *In silico* analysis of PpiA and PpiB proteins (182 and 308 amino acids, respectively) of *M. tb*, using protein analysis software (Lasergene Navigator; DNA STAR) displayed major antigenic stretches with a peak value ≥1.7. **(B)** Reactivity to rPpiA and rPpiB was studied then in two sets (patients and healthy groups) by ELISA. Statistical analysis of immunoactivity revealed that the patient group mounted significantly higher (*P* < 0.001) immune response against rPpiases compared to healthy controls. The horizontal line indicates the mean of the absorbance values.

His tagged *M. tb* rPpiA and GST tagged rPpiB were purified using Ni-NTA column and glutathione sepharose affinity column, respectively (Supplementary Figure 1). *In silico* antigenicity profiling of *M. tb* PpiA and PpiB protein (Figure 1A) using protein analysis software (Protean version 4.0, Lasergene Navigator; DNA STAR Inc; Madison, Wis) displayed major antigenic stretches with a peak value ≥1.7 (Chakhaiyar et al., 2004). To investigate if *M. tb* Ppiases could indeed elicit humoral immune response, experiments were designed to compare humoral immune response against the *M. tb* PpiA and PpiB in patients and healthy individuals. Statistical analysis revealed that TB patient group mounted a significantly higher (*P* < 0.001) antibody response against rPpiA and rPpiB compared to healthy group (Figure 1B). These results indicate that *M. tb* Ppiases elicit B-cell response and have possible immunomodulatory effect.

### rPpiases alter cytokine profile in THP-1 macrophages

**Figure 2:**
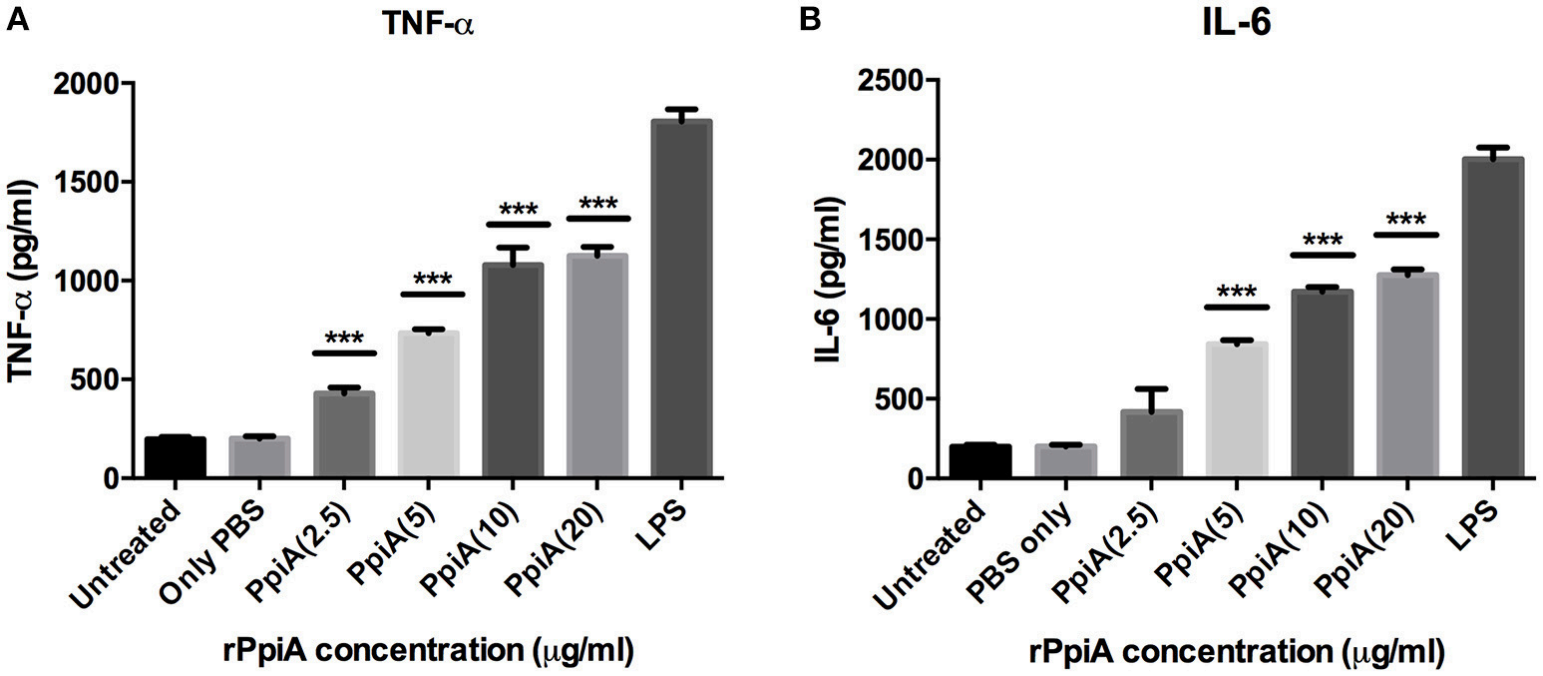
**PpiA stimulates secretion of pro-inflammatory cytokines by THP-1 cells**. Concentration dependent increase in the release of pro-inflammatory cytokines (**A**, TNF-α; and **B**, IL-6) as a function of treatment of THP-1 cells with rPpiA (2.5, 5.0, 10, and 20 μg) for 24 h. Data represent the mean ± *SD* of three replicates. LPS was used as a positive control. (^***^P < 0.001).

**Figure 3:**
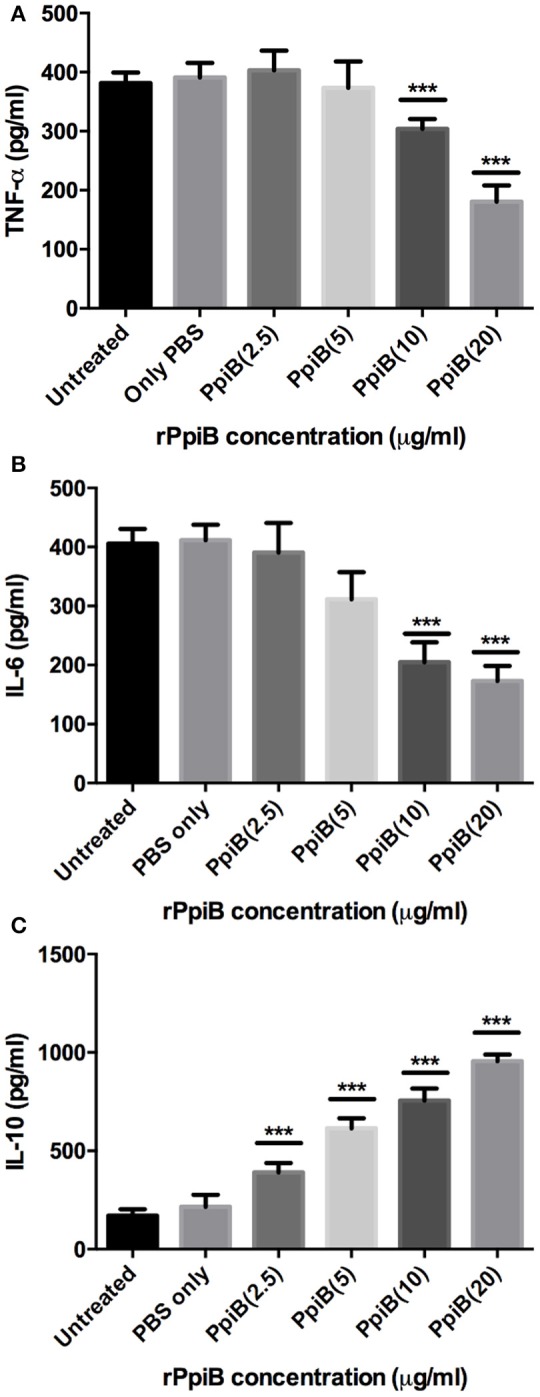
*****M. tb*** PpiB decreases secretion of TNF-α, IL-6 and stimulates secretion of IL-10 cytokine by THP-1 cells**. Dose dependent decrease in the release of pro-inflammatory cytokines (**A**, TNF-α; **B**, IL-6) and concomitant increase in the release of anti-inflammatory cytokine (**C**, IL-10), consequent to treatment by rPpiB (2.5, 5, 10, and 20 μg) for 24 h. Data represent the mean ± *SD* of three replicates. (^***^P < 0.001). LPS was used as positive control and mean levels of TNF-α and IL-6 observed in LPS treated cells were 1,753 ± 135 and 2070 ± 272, respectively.

Having shown the ability of *M. tb* Ppiases to elicit higher B-cell response, we investigated the ability of these proteins in modulating the secretion of various cytokines. Human monocytes (THP-1) differentiated with PMA were treated with increasing concentrations of polymyxin B treated rPpiA and rPpiB. Soup was collected after 24 h to estimate levels of different cytokines by ELISA. Significant increase in the levels of TNF-α and IL-6 (Figure 2) was observed, when treated with increasing concentration of rPpiA (2.5, 5, 10, 20 μg). However, no such significant increase in the levels of IL-10 could be noticed (Supplementary Figure 2). Interestingly, decrease in the level of TNF-α and IL-6 along with increase in the levels of IL-10 was observed (Figures 3A–C) when treated with increasing concentration of rPpiB (2.5, 5, 10, 20 μg). These results demonstrate that mycobacterial Ppiases alter cytokine secretion in human monocytic-macrophage cell lines. These observations are in agreement with previous reports involving mycobacterial chaperones and HSPs in immune modulation (Peetermans et al., 1994; Naffin-Olivos et al., 2014).

### *M. smegmatis* expressing *M. tb* Ppiases show increased survival under hydrogen peroxide stress

**Figure 4:**
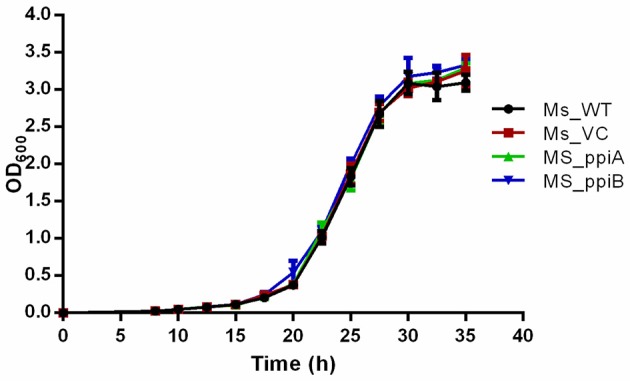
*****M. smegmatis*** carrying PpiA/PpiB do not alter bacterial growth ***in vitro*****. Bacterial growth (OD_600_) of wild type *M. smegmatis* (Ms_WT), *M. smegmatis* transformed with vector alone (Ms_VC), with vector carrying *M. tuberculosis ppiA* gene (Ms_ppiA) or *ppiB* gene (Ms_ppiB) was plotted. Data represent mean ± *SD* of values obtained from three independent cultures.

**Figure 5:**
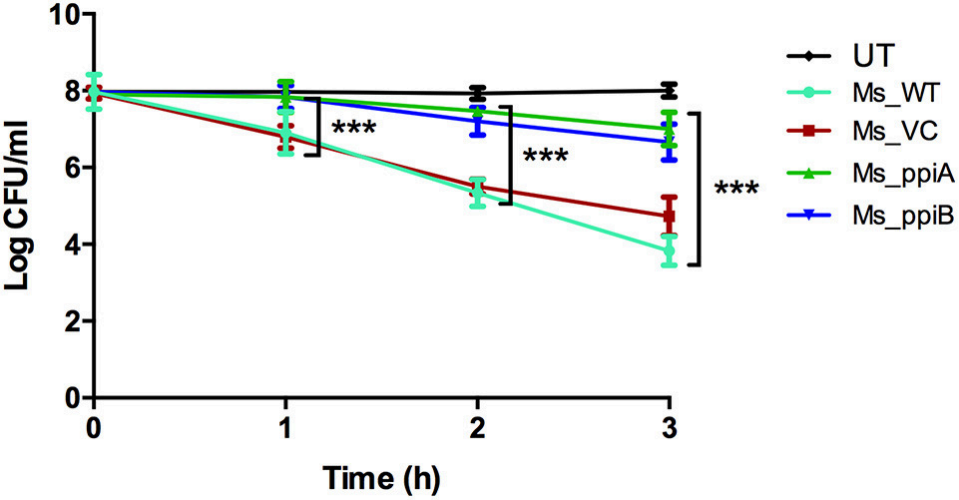
*****M. smegmatis*** growth profile under conditions of oxidative stress**. Secondary culture of wild type *M. smegmatis* mc^2^155, vector transformed *M. smegmatis*, or those transformed with vector plasmid carrying *M. tuberculosis ppiA* gene (Ms_ppiA), or *ppiB* gene (Ms_ppiB) were grown until O.D. = 0.4 and were treated with 7 mM H_2_O_2_ for 3 h and log c.f.u. was calculated at different time points. The data represent the mean ± SEM of triplicate wells and are representative of three individual experiments. (^***^P < 0.001).

*M. smegmatis* strains (Ms_WT, Ms_VC, Ms_ppiA, and Ms_ppiB) were grown in Middlebrook 7H9 broth with 10% OADC and growth profile was analyzed by measuring optical density at 600 nm. No significant difference was observed in the growth profile of recombinant *M. smegmatis* strains, as compared to the wild type *M. smegmatis* (Figure 4), indicating that overexpression of rPpiases does not affect *in vitro* growth of *M. smegmatis* under normal conditions. For investigating survival under hydrogen peroxide stress, which mimics one of the stresses faced by intracellular mycobacteria, log phase cultures (OD_600_ of 0.4) of recombinant *M. smegmatis* strains were treated with the 7 mM concentration of H_2_O_2_ for a period of 3 h. Colony forming unit counts indicated a significant difference in the survival of Ms_ppiA and Ms_ppiB as compared to the Ms_WT and Ms_VC cells (Figure 5). These results clearly indicate that *M. tb* cyclophilins play a critical role in stress adaptation, pointing to their role in virulence, as reported earlier, for another intracellular pathogen *Brucella abortus* (Roset et al., 2013).

### *M. smegmatis* expressing *M.tb* Ppiases show increased survival in human THP-1 cells

**Figure 6:**
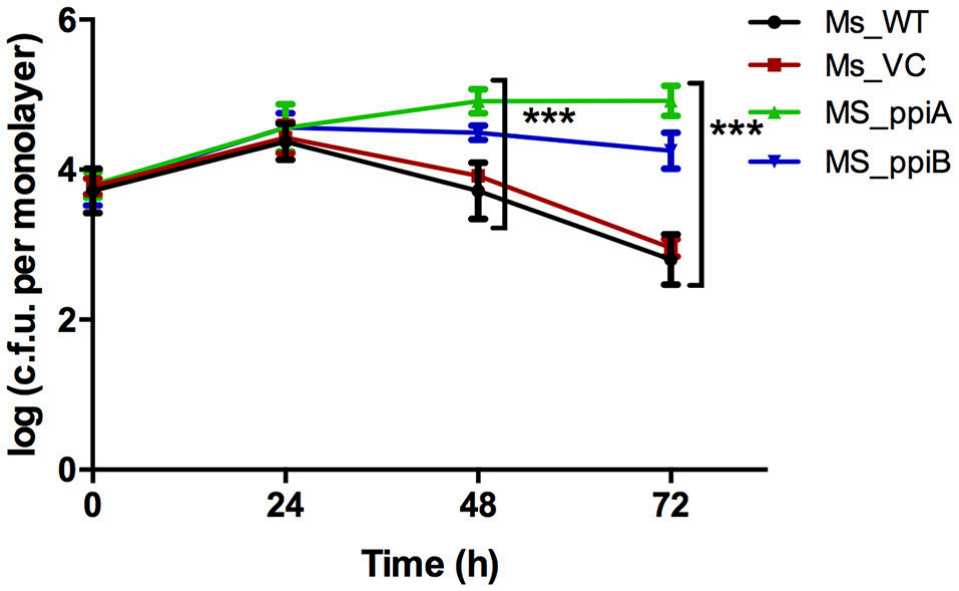
*****M. smegmatis*** expressing PpiA and PpiB show increased survival in THP-1 cells**. THP cells were infected with wild type *M. smegmatis* mc^2^155, vector control transformed *M. smegmatis*, or those transformed with *M. tuberculosis ppiA* gene (Ms_ppiA), and *ppiB* (Ms_ppiB) and plated after 24, 48, and 72 h and log c.f.u./monolayer was calculated at different time points. The data represented are mean ± SEM of triplicate wells and are representative of three individual experiments. (^***^P < 0.001).

To directly demonstrate the impact of *M. tb* PpiA and PpiB on survivability of mycobacterium in host cells, recombinant *M. smegmatis* strains were assayed for *in vitro* growth in THP-1 cells and compared with wild type *M. smegmatis*. As could be seen, recombinant Ms_ppiA and Ms_ppiB survived longer, upto 72 h (*p* < 0.001), in THP-1 cells than Ms_WT or Ms_VC (Figure 6). An increase of ~1 log was observed after 24 h in the control group, after which the colony forming unit (c.f.u.) declined. In contrast, Ms_ppiA and Ms_ppiB strains continued to replicate and survive up to 72 h within the macrophages. These results establish the critical role of Ppiases in intracellular survival.

## Discussion

Besides their biological function as helpers in protein folding, bacterial molecular chaperones have a distinctive role in virulence and stress tolerance. They are also termed as moonlighting proteins (Vanghele and Ganea, 2010). Hsp70 chaperone is present on bacterial surface and functions also as a plasminogen receptor in pathogens like *M. tb, Neisseria meningitides*, and *Listeria monocytogenes* (Knaust et al., 2007; Xolalpa et al., 2007). We investigated whether mycobacterial cyclophilins also have roles beyond protein folding to modulate the host immune response and assist in pathogenesis. Since B cells can exert influence on T cells, they are considered as important determinants in the outcome of infection with *M. tb*. *In-silico* analysis of *M. tb* PpiA and PpiB proteins displayed major antigenic stretches with a peak value ≥1.7. This was vindicated by the observation of significantly increased presence of antibodies to *M.tb* Ppiases in sera from TB patients as compared to healthy individuals. This is consistent with earlier findings that members of stress family of proteins such as Hsp70 and Hsp10 elicit strong B-cell response (Mehlert and Young, 1989; Young and Garbe, 1991). These findings clearly indicate the immunodominant nature of these cyclophilins expressed during infection and also highlight their likely diagnostic potential.

In *M. tb* infection cycle, macrophages act as the first line of defense and the pathogen hires a plethora of strategies to counteract the host immune response. Skewing of the balance in secretion of pro-inflammatory and anti-inflammatory cytokines results in intracellular bacterial clearance or survival, respectively (Newman et al., 1991; Byrd, 1997). Chronic inflammatory pathology is the hallmark of tuberculosis, which indicates that overproduction of pro-inflammatory cytokines lies at the heart of the infection. TNF-α is crucial for the formation and maintenance of granuloma that are main effector sites of antimicrobial activity against the pathogen (Kindler et al., 1989). Estimation of proinflammatory cytokines secreted by THP-1 cells on treatment with increased doses of rPpiA revealed that this Ppiase is a potent stimulator of TNF-α and IL-6 and thus may play an important role in host inflammatory pathology. Mycobacterial HSPs Hsp65 and chaperones have been earlier shown to act as strong immune modulators and are potent stimulators of pro-inflammatory cytokines (Peetermans et al., 1994; Naffin-Olivos et al., 2014).

**Figure 7:**
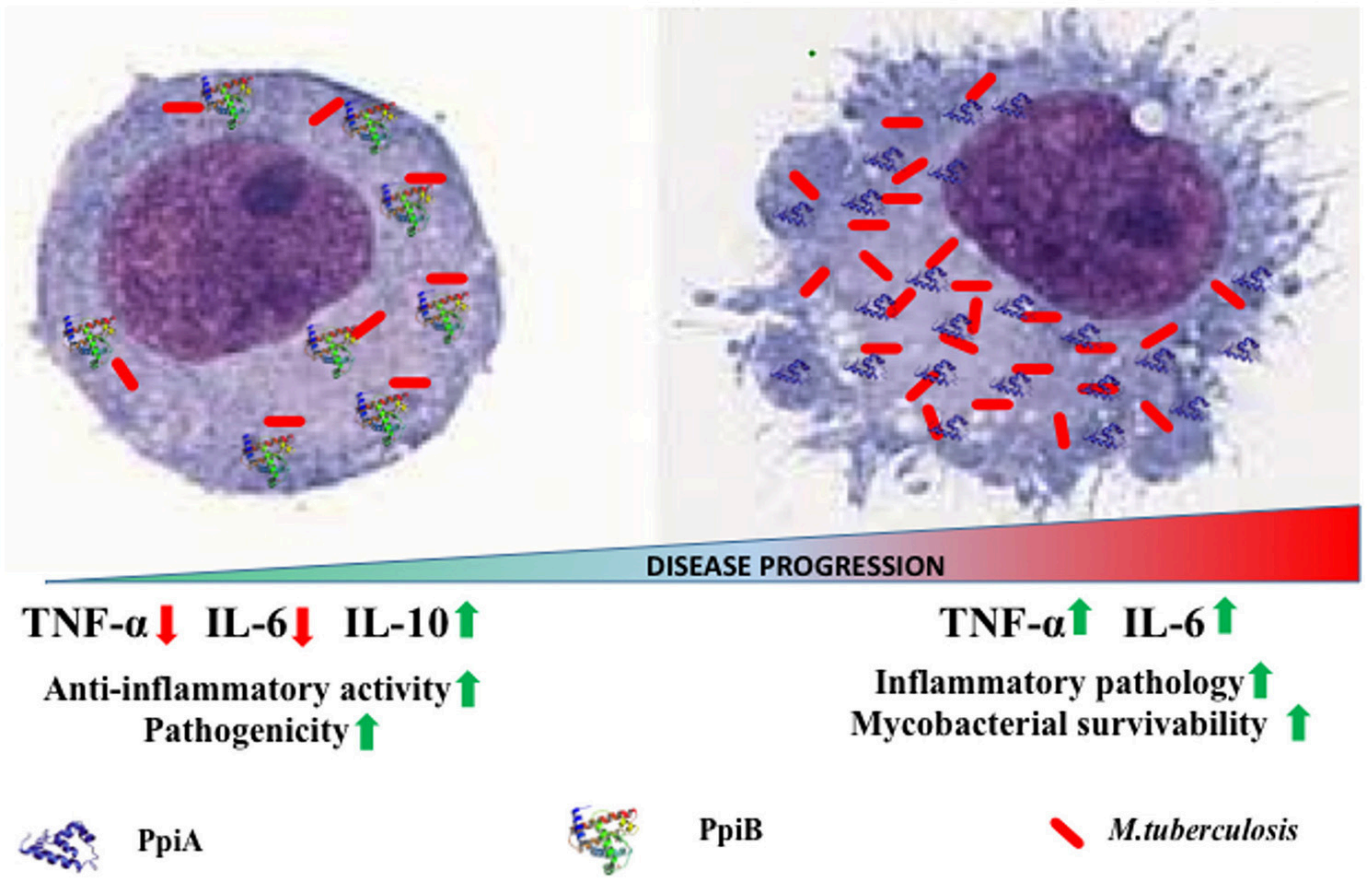
**Model of PpiA and PpiB action on ***M. tb*** pathogenesis**. PpiB aids mycobacterial growth by production of inhibitory cytokine IL-10. PpiA induces secretion of pro-inflammatory cytokines and may be associated with immunopathological responses in tuberculosis, which aids in bacterial dissemination and disease progression.

Our results implicate *M. tb* PpiA as a stimulator of pro-inflammatory cytokines thereby pointing to its vital role in the inflammatory pathology of tuberculosis (Figure 7). Conversely, assessment of pro-inflammatory cytokines secreted by THP-1 cells upon treatment with increasing concentrations of rPpiB revealed that the protein is an inhibitor of TNF-α and IL-6 along with stimulating secretion of IL-10. That *M. tb* PpiB is able to subvert the innate immune response points to its role in aiding the establishment of infection, which fits well with previous findings that PpiB is essential for the survival of the pathogen (Sassetti et al., 2003).

The kinetics of production along with balance between pro-inflammatory and anti-inflammatory cytokines released by macrophages upon exposure to mycobacterial antigens regulate the T cell responses (Dheenadhayalan et al., 2006; Kim et al., 2011). Production of pro-inflammatory cytokines is known to contribute to the host response against mycobacteria (Ladel et al., 1997a; Tsao et al., 1999; Sasindran and Torrelles, 2011). Our apparently paradoxical observation that PpiA increases intracellular survival despite the pro-inflammatory role suggests an alternative role of PpiA mediated TNF-α and IL-6. Since PpiA is known to be expressed late in the infection cycle (Pathakumari et al., 2015), the pro-inflammatory cytokines induced by PpiA is likely associated with immunopathological responses in tuberculosis leading to necrosis and cachexy that aid in disease progression. Production of TNF-α has earlier been shown to be directly related to virulence correlating with intracellular viability (Newman et al., 1991; Engele et al., 2002) and is also known to promote growth of virulent *M. tb* in monocytes (Byrd, 1997). Thus, induction of PpiA mediated TNF-α by macrophages at the site of infection permits the multiplication of intracellular bacteria and may therefore present an evasion mechanism employed by *M. tb*. This pro-inflammatory response induced by high bacterial load is also known to induce apoptosis that is associated with mycobacterial survival (Santucci et al., 2000). Earlier studies have also shown that mycobacterial components increase the production of pro-inflammatory cytokine TNF-α in macrophages/monocytes and this elevated level of TNF-α is regarded as reason for mycobacterial persistence and virulence within human macrophages (Dheenadhayalan et al., 2006; Kim et al., 2011). IL-6, although important for control of tuberculosis in mice model (Ladel et al., 1997b), in humans is considered as a correlate of disease progression because of its role in inflammation and pathology (Tsao et al., 1999; Ilonidis et al., 2006). Conversely, PpiB employs the classical approach to aid mycobacterial growth by production of inhibitory cytokine IL-10 (Figure 7). The different strategies employed by the same classes of protein is possibly a function of the difference in their expression kinetics: PpiB being expressed early during infection employs IL-10 to dampen the inflammatory host response, whereas PpiA, expressed late in infection (Pathakumari et al., 2015) aids in immunopathology. IL-10 secreted by host cell after mycobacterial infection is also known to increases the intracellular bacterial survivability by blocking phagosomal maturation (O'Leary et al., 2011). Although Ppiases are constitutively expressed for their action as a peptidyl prolyl isomerase to catalyze the cis-trans isomerization of proline imidic peptide bonds in oligopeptides, the convergent evolution has adapted the mycobacterial proteins for moonlighting functions. Thus, different immunological response could be based upon the different expression patterns at different stages of infection cycle. Although currently there are no gene expression data to claim this but an earlier study wherein PpiA was shown to induce IFN-γ in LTBI as compared to that of PTB suggested enhanced expression of this protein, late in infection cycle (Pathakumari et al., 2015). Moreover PpiA is also known to be upregulated in intraphagosomal niche during infections (Mattow et al., 2006). On the contrary PpiB being essential for the *in vitro* growth of *M. tb*, suggests its expression early during infection.

Intracellular infectious agents that have co-evolved in long standing association with the host have acquired mechanisms to persist within the host cell. The persistence of pathogenic mycobacteria within the hostile environment of host macrophages is in part due to the bacterial ability to adapt to the stress conditions encountered. Our data suggest that *M*. *tb* Ppiases might play a role in the intracellular survival by subverting the host cell defenses, such as oxidative stress as well as by immunomodulation. *M*. *tb* Hip1 has been shown to modulate macrophage responses through proteolysis of GroEL2 (Naffin-Olivos et al., 2014) and this augers well with our earlier report of chaperone activity of *M*.*tb* Ppiases (Pandey et al., 2016), though the exact mechanism remains to be elucidated. In conclusion, our study demonstrates immunomodulatory potential of *M. tb* Ppiases and in the process unveils previously unknown functions of peptidyl-prolyl isomerases. The novel immunological features attributed herein to PpiB, an essential protein of *M. tb*, highlights the importance of this protein/enzyme as an important target for the development of more efficacious therapeutic interventions against TB.

## Ethics statement

This study was carried out in accordance with the recommendations of Institutional Bioethics Committee, Bhagwan Mahavir Medical Research Centre, Hyderabad, India with written informed consent from all subjects. All subjects gave written informed consent in accordance with the Declaration of Helsinki. The protocol was approved by the Institutional Bioethics Committee.

## Author contributions

NE and SH conceptualized and designed the research; SP, DT, and MK performed research; SP, DT, AK, JS, SH, and NE carried out data analysis; GS recruited and classified human subjects; SP, DT, JS, SH, and NE wrote the manuscript.

## Funding

This work was partially funded by a Centre of Excellence research grant (BT/PR12817/COE/34/23/2015) to SH and NE from the Department of Biotechnology, Ministry of Science and Technology (DBT), Government of India.

### Conflict of interest statement

The authors declare that the research was conducted in the absence of any commercial or financial relationships that could be construed as a potential conflict of interest.
