# Supplementary material for: Mycobacterium tuberculosis Peptidyl-Prolyl Isomerases Are Immunogenic, Alter Cytokine Profile and Aid in Intracellular Survival
Source: Front Cell Infect Microbiol. 2017 Feb 15;7:38. doi: 10.3389/fcimb.2017.00038 (PMC5310130; doi:10.3389/fcimb.2017.00038)
Supplement: Supplementary file 1 [file Table1.DOCX]

Supplementary Material

***Mycobacterium tuberculosis* peptidyl-prolyl isomerases are immunogenic, alter cytokine profile and aid in intracellular survival**

Saurabh Pandey, Deeksha Tripathi, Mohd. Khubaib, Ashutosh Kumar, Javaid Ahmad Sheikh, Gaddam Sumanlatha, Nasreen Zafar Ehtesham^*^, Seyed Ehtesham Hasnain^*^

*** Correspondence:**

Nasreen Zafar Ehtesham Email: nzehtesham@gmail.com

Seyed Ehtesham Hasnain Email: seyedhasnain@gmail.com

# Supplementary Table

**TABLE S1. Strains and plasmids used in this study**

**Plasmids Relevant characteristics Source/Reference**

pET28_ppiA pET28a containing *ppiA*, *kan^R^** This work

pGEX_ppiB pGEX6p1 containing *ppiB*, *amp^R^** This work

pST_ppiA pST_2K containing *ppiA*, *kan^R^** This work

pST_ppiB pST_2K containing *ppiB*, *kan^R^** This work

* Selection marker resistant to Ampicillin (*amp^R^*) and kanamycin (*kan^R^*).

**Strains Relevant characteristics Source/Reference**

DH5α *supE44* *ΔlacU(Φ80lacZΔM*1*5) hsdR*1*7 rec*1 *endA*1 *gyrA96 thi-*1 *relA*1 Novagen

BL-21(DE3) *F– ompT gal dcm lon hsdSB(rB- mB-) λ (DE3 [lacI lacUV5-T7 gene* 1 Novagen

Ms_WT *M. smegmatis* mc2 155 ATCC

Ms_VC *M. smegmatis* containing pST-2K vector This work

Ms_ppiA *M. smegmatis* containing pST-ppiA This work

Ms_ppiB *M. smegmatis* containing pST-ppiB This work
