# Supplementary material for: Mycobacterium tuberculosis Peptidyl-Prolyl Isomerases Are Immunogenic, Alter Cytokine Profile and Aid in Intracellular Survival
Source: Front Cell Infect Microbiol. 2017 Feb 15;7:38. doi: 10.3389/fcimb.2017.00038 (PMC5310130; doi:10.3389/fcimb.2017.00038)
Supplement: Supplementary file 2 [file Table2.DOCX]

***Supplementary Material***

***Mycobacterium tuberculosis* peptidyl-prolyl isomerases are immunogenic, alter cytokine profile and aid in intracellular survival**

Saurabh Pandey, Deeksha Tripathi, Mohd. Khubaib, Ashutosh Kumar, Javaid Ahmad Sheikh, Gaddam Sumanlatha, Nasreen Zafar Ehtesham^*^, Seyed Ehtesham Hasnain^*^

*** Correspondence:**

Nasreen Zafar Ehtesham Email: nzehtesham@gmail.com

Seyed Ehtesham Hasnain Email: seyedhasnain@gmail.com

**Supplementary Table**

**Table S2.** List of Primers used in the study.

**Primer Sequence Restriction Enzyme**

PpiA_ F CGGGATCCATGGCAGACTGTGATTCCGTGACTAA *Bam*HI

PpiA_R GGGAAGCTTTCAGGAGATGGTGATCGACTCGATC *Hin*dIII

PpiB_ F CGGGATCCATGGGCCACTTGACACCG *Bam*HI

PpiB_R GGGAAGCTTCTAATCCAGCAGCACCGACGTG *Hin*dIII
