# Supplementary material for: Mycobacterium tuberculosis Peptidyl-Prolyl Isomerases Are Immunogenic, Alter Cytokine Profile and Aid in Intracellular Survival
Source: Front Cell Infect Microbiol. 2017 Feb 15;7:38. doi: 10.3389/fcimb.2017.00038 (PMC5310130; doi:10.3389/fcimb.2017.00038)
Supplement: Supplementary file 3 [file Image1.PDF]

Supplementary Material

Mycobacterium tuberculosis peptidyl-prolyl isomerases are immunogenic, alter cytokine profile and aid in intracellular survival

Saurabh Pandey, Deeksha Tripathi, Mohd. Khubaib, Ashutosh Kumar, Javaid Ahmad Sheikh, Gaddam Sumanlatha, Nasreen Zafar Ehtesham*, Seyed Ehtesham Hasnain*

*** Correspondence:**

Nasreen Zafar Ehtesham Email: nzehtesham@gmail.com

Seyed Ehtesham Hasnain Email: seyedhasnain@gmail.com

Supplementary Figure

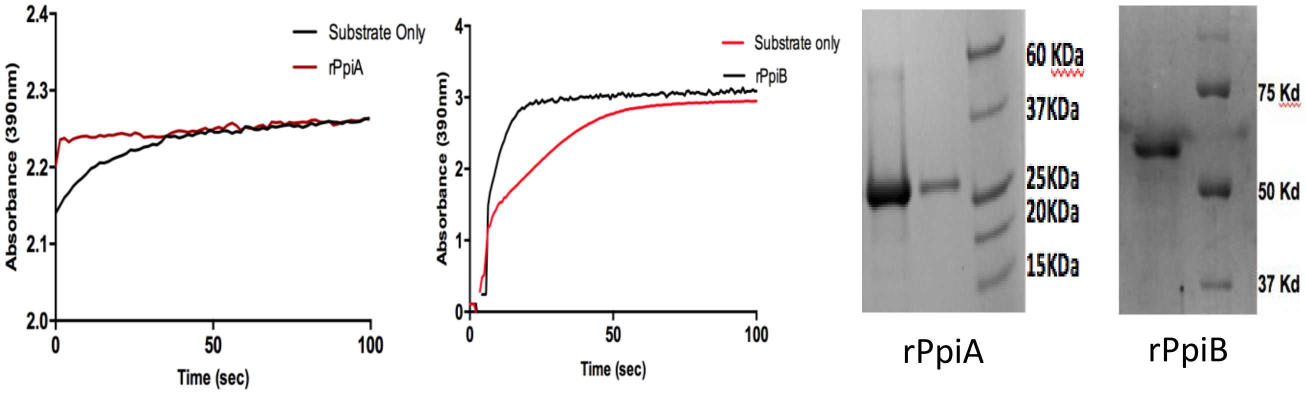

Supplementary Figure 1: Recombinant Ppiases proteins are enzymatically active. His tagged *M. tb* rPpiA and GST tagged rPpiB were purified using Ni-NTA column and glutathione sepharose affinity column, respectively. rPpiA displayed the expected 25kDa molecular size while rPpiB protein band was observed at 69kDa molecular size on SDS PAGE (Pandey et al., 2016). Enzymatic activity of the purified proteins was measured, as shown earlier (Pandey et al., 2016) in a spectrophotometric-coupled assay using the chromogenic peptide N-succinyl-Ala-Ala-Pro-Phe-p-nitro-anilide and α-chymotrypsin at 15°C. Increase in the rate of isomerization activity, as compared to the control, demonstrate that the recombinant Ppiases are enzymatically active (Henriksson et al., 2004).
