# Supplementary material for: Mycobacterium tuberculosis Peptidyl-Prolyl Isomerases Are Immunogenic, Alter Cytokine Profile and Aid in Intracellular Survival
Source: Front Cell Infect Microbiol. 2017 Feb 15;7:38. doi: 10.3389/fcimb.2017.00038 (PMC5310130; doi:10.3389/fcimb.2017.00038)
Supplement: Supplementary file 4 [file Image2.PDF]

Supplementary Material

Mycobacterium tuberculosis peptidyl-prolyl isomerases are immunogenic, alter cytokine profile and aid in intracellular survival

Saurabh Pandey, Deeksha Tripathi, Mohd. Khubaib, Ashutosh Kumar, Javaid Ahmad Sheikh, Gaddam Sumanlatha, Nasreen Zafar Ehtesham*, Seyed Ehtesham Hasnain*

*** Correspondence:**

Nasreen Zafar Ehtesham Email: nzehtesham@gmail.com

Seyed Ehtesham Hasnain Email: seyedhasnain@gmail.com

Supplementary Figure

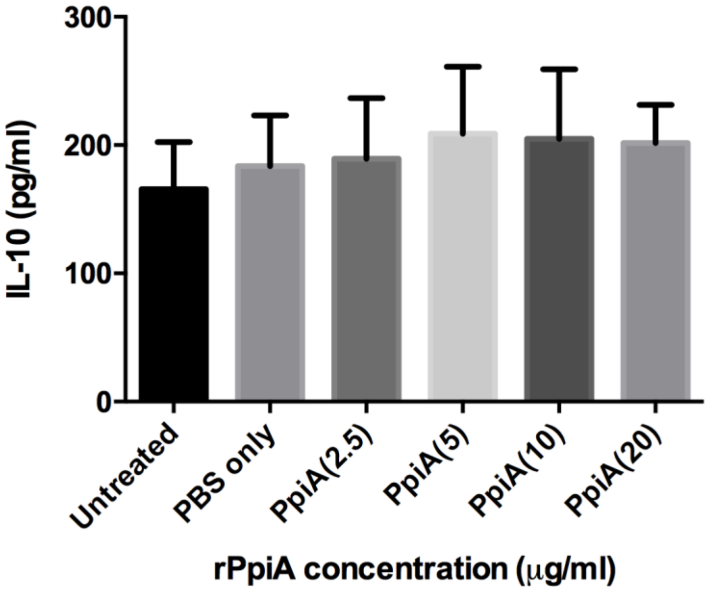

Supplementary Figure 2. PpiA stimulation does not affect secretion of IL-10 cytokines by THP-1 cells.
